# Hospitalization due to mental and behavioral disorders caused by use of alcohol and psychoactive substances among older adults and elderly people in Brazil: a cross-sectional study

**DOI:** 10.1590/1516-3180.2021.0115.R1.22062021

**Published:** 2022-03-14

**Authors:** Pedro Paulo Luciano Afonso, Mariana Luciano Afonso, Gabriela Rodrigues Barbosa, Alberto Fernando Oliveira Justo

**Affiliations:** I MD. Resident of Psychiatry, Department of Psychiatry, Hospital Municipal do Campo Limpo, São Paulo (SP), Brazil.; II PhD. Psychologist, Assistant Professor, Department of Medicine, Universidade Cidade de São Paulo (UNICID), São Paulo (SP), Brazil; III MSc. Biomedical Scientist and Doctoral Student, Department of Medicine, Universidade Federal de São Paulo (UNIFESP), São Paulo (SP), Brazil.; IV PhD. Biomedical Scientist and Associate Researcher, Department of Medicine, Universidade Federal de São Paulo (UNIFESP), São Paulo (SP), Brazil.

**Keywords:** Alcohol-induced disorders, Substance-related disorders, Aging, Psychiatric conditions, DATASUS, CAPS

## Abstract

**BACKGROUND:**

It has been estimated that 17% of individuals aged 50 years or older suffer from addiction to legal or illegal drugs. Use of alcohol and psychoactive substances has been correlated with several diseases, e.g. psychiatric conditions and cardiovascular and sexual dysfunctions.

**Objective:**

To discuss the Brazilian profile of mental and behavioral disorders caused by use of alcohol and psychoactive substances among older adults and elderly people, over the period from 2008 to 2019.

**DESIGN AND SETTING:**

Cross-sectional study conducted among Brazilians aged 50 years or older.

**METHODS:**

Hospitalization due to mental and behavioral disorders caused by use of alcohol and psychoactive substances was assessed through data obtained from the National Health System Department of Informatics (Departamento de Informática do Sistema Único de Saúde, DATASUS).

**RESULTS:**

Decreasing and steady trends of hospitalization due to mental and behavioral disorders caused by use of alcohol among both men and women at all ages were observed. Similar trends were reported for all age ranges among men and women aged 60 years and older. In contrast, a slight increase was seen among women aged 50 to 59 years.

**CONCLUSION:**

These data are crucial for qualifying mental healthcare for older adults and elderly people and for planning mental health services.

## INTRODUCTION

Drug addiction is a common issue reported among adolescents and young adults. However, it has been estimated that 17% of individuals aged 50 years old or older suffer from addiction to legal or illegal drugs.^1,2^ There is evidence from several studies showing that sexual and psychological abuse, maltreatment and trauma during childhood are closely related to substance use in later years.^3,4^

Accumulating evidence has shown that significant levels of psychological distress are present among older adults and elderlies. A previous study^5^ showed that there was an increasing rate of hospitalization due to drug substance abuse and a decreasing rate due to alcohol among older adults and elderly people in the United States from 1992 to 2005. In addition, increasing rates of first hospitalization due to substance abuse were reported among older adults and elderly people from 1998 to 2008.^6^ Increasing suicide rates were reported among women and men aged up to 74 years from 2000 to 2014, and the rate is expected to increase further over the next few years.^7^

Among older adults and elderly people, the symptoms of substance abuse are more challenging to treat and may be consequences of other diseases, e.g. cardiovascular diseases,^8^ urological diseases,^9^ diabetes^10^ and cancer.^11^ There are also significant relationships between abusive use of alcohol and psychoactive substances and occurrence of mental and behavioral disorders. For example, in a cross-sectional study on the clinical characteristics of drug users hospitalized in an intensive care unit, it was found that 31.2% of the individuals had psychiatric comorbidities, and that the most common of these were depression, anxiety and bipolar affective disorder.^12^ In the psychological and psychiatric literature, it is indicated that abusive use of alcohol and drugs can act both as an aggravator and as a consequence of mental illness.^13,14^

Alcohol and substance abuse disorders are a risk factor for several diseases, e.g. psychiatric conditions, cardiovascular conditions and sexual dysfunctions, and have become a public health issue that potentially affects individuals’ lives, employment and family relationships.^15,16^ One explanation for this the influence of cultural factors that normalize alcohol abuse and facilitate increasing alcohol consumption, as seen in Brazil. Another important explanation is that alcohol and substance abuse negatively affect family relationships. Thus, conflict-ridden, violent and unstructured family relationships act as a predisposing factor for alcohol abuse. A similar dynamic can be observed in relation to work: while it is true that alcohol and substance abuse affects working relationships, it is also true that frustration in labor relations and unemployment are also factors that give rise to vulnerability to abusive use of alcohol and substances.^17^ Cumulative studies^18–20^ within the fields of social psychology and occupational medicine have shown that the organizational structure of work relationships can lead individuals to emotional distress, which can turn into mental health issues such as depression, stress and anxiety. Furthermore, one common consequence of depression is increased consumption of alcohol and substances.^19,21^

Treatment of alcohol and substance abuse remains challenging. It is important to mention the difficulties reported by healthcare service professionals, such as the difficulty of establishing clear criteria for discharge processes for individuals with substance abuse issues.^22^ These difficulties relate to the following: divergences in professional teams; omnipotence of professionals; difficulties relating to rupture of bonds; institutional dependency; patient instability; and difficulties in making connections within the healthcare network. Previous research has indicated that the treatment and discharge process is complex and is characterized by combinations of social, economic, political, subjective and institutional dimensions.^22^

The National Household Sampling Survey (Pesquisa Nacional por Amostra de Domicílios, PNAD) investigates the general characteristics of the Brazilian population and its living conditions every year. It includes health-related questions every three years, and accesses individual information on demographic and socioeconomic characteristics and selected health indicators, including risk factors and self-reported chronic diseases. According to PNAD, less than 10% of the subjects who reported having alcohol and drug-related problems have received treatment for their disorders.^23^ Understanding the national profile of individuals who need treatment for alcohol and substance abuse is a key point for planning public policies and improving the quality of treatment for these disorders.^17^

In this regard, cross-sectional studies on mental health are an important tool for analyzing the distribution and frequency of mental disorders. Such studies contribute to planning, executing and evaluating strategic interventions for prevention, control and treatment.

## OBJECTIVE

Since mental health during the aging process and use of psychoactive drugs and alcohol in later life are still unrecognized problems, the aim of the present study was to analyze hospitalization due to mental and behavioral disorders related to alcohol and psychoactive substance abuse in Brazil over the period from 2008 to 2019.

## METHODS

### Clinical data

This cross-sectional study was conducted in September 2020. It included data from hospital admissions that were reported as mental and behavioral disorders due to use of alcohol or psychoactive substances among individuals aged 50 years or older who were living in Brazil. The period surveyed was from January 1, 2008, to December 31, 2019.

The data were extracted from the database of the Department of Informatics of the Brazilian National Health System (Departamento de Informática do Sistema Único de Saúde, DATASUS), which is part of the Ministry of Health (Ministério da Saúde, MS) and is available through the DATASUS website.^24^ The data available through DATASUS are part of the universal accessibility policy of the Brazilian public healthcare system and include the hospital information system, which is composed of the registers collected through municipal health departments. The data collection methodology did not change during the study period.

The subjects whose information was extracted were not individually identified. Therefore, this study did not require approval from a research ethics committee.^25^

The information in DATASUS includes the basic and associated cause based on the 10^th^ edition of the International Classification of Diseases (ICD). We used the ICD version 10 (ICD-10) codes, among which F10 corresponds to mental and behavioral disorders due to use of alcohol and F11 to F19 correspond to mental and behavioral disorders due to psychoactive substance use, which includes use of opioids, cannabinoids, sedative-hypnotics, cocaine, other stimulants such as caffeine, hallucinogens and volatile solvents and multiple drug use of other psychoactive substances.

### Demographic data

Demographic data were obtained from the Brazilian Institute for Geography and Statistics (Instituto Brasileiro de Geografia e Estatística, IBGE).^26^ The IBGE is the official provider of geographical and statistical information in Brazil, and it conducts a census every 10 years to verify the profile of the Brazilian population by collecting several variables from every household in the country. The sociodemographic profile in the years between censuses are estimated through projections.

### Data analysis

Data analyses were conducted using the Prism software, version 6.0 (GraphPad Software, San Diego, California, United States). For analyses on the differences across age groups, the one-way analysis of variance (ANOVA) test was used as described previously.^27^ The statistical tests were considered significant when P < 0.05.

For the proportional rates of hospitalization, we used the total number of hospitalizations according to age as the numerator, and the respective population as the denominator, as shown in the following equation:

$${\mathrm{Hospitalization}}_{\mathrm{age}}=\frac{\text{number of hospitalizations}}{\text{total population in the range}}\times 10^{5}$$

## RESULTS

The aim of this epidemiological study was to evaluate the hospitalization profile of mental and behavioral disorders due to use of alcohol and psychoactive substances among Brazilians aged 50 years and older.

Between 2008 and 2019, 184,930 individuals were hospitalized in Brazil due to mental and behavioral disorders caused by alcohol. Interestingly, the hospitalization rate was nine times higher among males than among females. In both genders, individuals aged 50 to 59 years were the ones most affected, followed by the age ranges of 60 to 69 years, 70 to 79 and 80 or older **(Table 1)**.

**Table 1.** Frequency of hospitalization due to mental and behavioral disorders caused by psychoactive substance use among Brazilians between 2008 and 2019

Age (years)	Men		Women	
	n	% (95% CI)	n	% (95% CI)
50-59	18,503	77.98 (78.50 ± 77.44)	5,454	69.40 (70.42 ± 68.37)
60-69	3,923	16.53 (17.01 ± 16.06)	1,478	18.80 (19.69 ± 17.95)
70-79	947	3.99 (4.25 ± 3.74)	613	7.81 (8.41 ± 7.21)
≥ 80	355	1.50 (1.65 ± 1.34)	313	3.99 (4.43 ± 3.56)
**Total**	**23,728**	**100**	**7,858**	**100**

Data source: Hospital Information System, available from the Department of Informatics of the Brazilian National Health System. CI = confidence interval.

**Figure 1.**
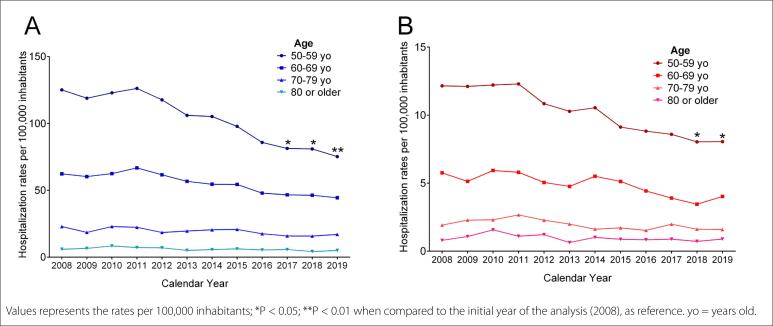
Proportional hospitalization due to mental and behavioral disorders caused by use of alcohol (International Classification of Diseases version 10, ICD-10: F10) among males (A) and females (B) according to their respective populations.

In order to analyze the real rates of hospitalization, considering the demographic increase of the population, we cross-referenced the demographic data and hospital admission registers over the period studied. In proportional terms, males **(Figure 1A)** showed massively higher rates of hospitalization, compared with females **(Figure 1B)**. However, the two sexes presented similar trends, with significantly decreasing rates (P < 0.05) among individuals aged 50 to 59, a slight decrease among those aged 60 to 69 years and steady rates among those aged 70 years and older.

Over the same period, 31,586 Brazilians were hospitalized due to mental and behavioral disorders caused by psychoactive substance use. Among these individuals, the hospital admission rate for men was approximately three times higher than the rate for women. Again, comparing the age ranges within the genders, there were similar profiles: male and female individuals aged 50 to 50 were the ones most hospitalized, followed by those aged 60 to 69, 70 to 79 and 80 or older **(Table 2)**.

**Table 2.** Frequency of hospitalization due to mental and behavioral disorders caused by alcohol use among Brazilians between 2008 and 2019

Age (years)	Men		Women	
	n	% (95% CI)	n	% (95% CI)
50-59	120,502	72.19 (72.40 ± 71.96)	12,895	71.65 (72.31 ± 70.99)
60-69	38,926	23.32 (23.52 ± 23.11)	3,991	22.19 (22.79 ± 21.57)
70-79	6,667	3.99 (4.08 ± 3.90)	887	4.91 (5.25 ± 4.61)
≥ 80	839	0.50 (0.53 ± 0.46)	223	1.23 (1.41 ± 1.08)
**Total**	**166,934**	**100**	**17,996**	**100**

Data source: Hospital Information System, available from the Department of Informatics of the Brazilian National Health System. CI = confidence interval.

**Figure 2.**
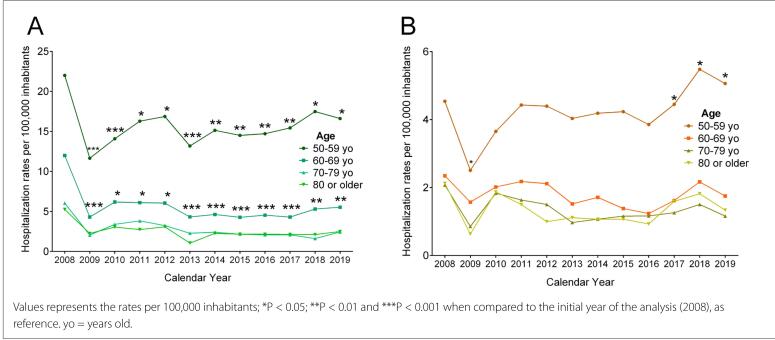
Proportional hospitalization due to mental and behavioral disorders caused by psychoactive substance use For Review Only (International Classification of Diseases version 10, ICD-10: F11-F19) among males (A) and females (B) according to their respective populations.

In proportional terms, hospitalization among males was always higher. At all ages, there was a slight decrease in the rates

(P > 0.05) **(Figure 2A)**. However, among females, a statistical increase (P < 0.05) was reported among those aged 50 to 59 years, while a steady rate at all ages from 60 years onwards was observed **(Figure 2B)**.

Lastly, comparing hospitalization due to alcohol with hospitalization due to psychoactive substances (Tables 1 and 2), the hospitalization rate for mental and behavioral disorders due to alcohol abuse was approximately six times higher (184,930 individuals) than for psychoactive substances (31,586 individuals).

## DISCUSSION

In the present epidemiological study, we analyzed the hospitalization profile of mental and behavioral disorders due to use of alcohol and psychoactive substances in Brazil over the period 2008-2019. We also evaluated their prevalence according to sex, age ranges and calendar-year incidence of hospitalization. Over the period studied, we observed that the hospitalization rate for mental and behavioral disorders due to alcohol abuse was approximately six times higher than the rate due to psychoactive substances. In addition, men were hospitalized more, due to both use of alcohol and use of psychoactive substances. Our results showed that hospital admission due to alcohol use has been decreasing in both sexes aged 50 to 69 years, while a steady rate profile was observed among individuals aged 70 years and older. Different profile was seen with regard to hospitalization due to psychoactive substances; females aged 50 to 59 years presented an increasing rate over the years, while steady rates were observed among females aged 60 years and older and at all ages among men.

According to the World Health Organization (WHO),^28^ abuse of alcohol results in approximately three million deaths per year (5% of all deaths worldwide). This mortality rate is higher than the rates for diseases such as diabetes, tuberculosis and human immunodeficiency virus/acquired immunodeficiency syndrome (HIV/AIDS). In 2016, 133 million cases of alcohol-attributable disability were reported worldwide. Among these disabilities induced by alcohol abuse, 49% were due to non-communicable and mental health conditions, 40% due to accidents and 11% due to digestive, cardiovascular and infectious diseases, and to cancers. In Brazil, about 9% of all disabilities are related to mental and behavioral disorders, according to the Brazilian Ministry of Labour.^29^

It is widely known that alcohol consumption is more prevalent among males than among females. Among all deaths, 7.7% of those among males and 2.6% of those among females are related to alcohol worldwide.^30^ Our results showed that hospitalization due to mental and behavioral disorders caused by use of alcohol was nine times higher among males. Drinking habits can be influenced by several factors, e.g. demographic, social and attitudinal variables.^31^ One plausible explanation for the higher levels of drinking among men relates to the traditional form of masculinity, in which being a man and consumption of alcohol are strongly associated. The massive difference between men and women can also be explained by the fact that drinking behavior has traditionally been condemned by women. In a survey conducted recently among young men in Brazil, there was a positive correlation between alcohol consumption and adherence to these traditional norms of masculinity^32^ (such as heterosexuality, aggressiveness and a tendency to engage in risky behavior,^33,34^ which reinforces our point of view. It is also important to point out that the conventional forms of masculinity are closely associated with the worst health outcomes.^35^

Addiction to legal and illegal drugs is commonly an issue related to early adulthood^36^ and adolescence.^37^ Interestingly, consumption of drugs tends to decrease in later adulthood (approximately at the age of 50 years) in both genders,^38^ with the exception of tobacco. In our results, we showed that there were higher rates of hospitalization due to alcohol use in the younger population in both genders. According to a study conducted by the Brazilian government,^39^ 8% of Brazilians aged 45 to 59 years old consume alcohol every day, 16% up to four times per week, 14% up to three times per month, 9% up one time per month and 53% less than once per year or have never used alcohol. Among elderly people aged 60 years or older, 7% drink alcohol every day, 8% up to four times per week, 10% up to three times per month, 8% up to one time per month and 68% have never drunk alcohol or less than once per year. In Brazil, 50,296 psychiatric hospital admissions due to alcohol use were reported in 2010 and 34,249 in 2017, which was a decrease of about 32%.^40^ We attributed the decline in hospitalization to: i) efficient implementation and popularization of centers for psychosocial care (Centros de Atenção Psicossocial, CAPS) in Brazil; and ii) culturally, drinking habits are lower in the older population.

A previous study^41^ showed that the use of illicit drugs among individuals aged 50 to 59 years old increased from 5.1% in 2002 to 9.4% in 2007, and 90% of this population started using them when they were younger than 30 years old. In the United States, it was estimated that in 2000, 1.7 million people aged 50 years or older needed treatment due to drug abuse, and 4.4 million in 2020.^42^

In this study, we showed that hospitalization due to psychoactive substance use (which includes use of opioids, cannabinoids, sedative-hypnotics, cocaine, other stimulants such as caffeine, hallucinogens and volatile solvents and multiple drug use of other psychoactive substances) and due to alcohol is approximately three and nine times higher, respectively, among men than among women. One key point in this scenario is that recreational use of psychoactive substances and alcohol is more condemned among women, which may be reflected in the lower rates of hospitalization among females.

In addition to the cultural factors that influence the differences in the levels of alcohol and psychoactive substance abuse among men and women, another cultural factor that is very influential within the Brazilian reality is religiosity. It is important to highlight the great influence of religion in Brazil and its role in the use of alcohol and psychoactive substances. Self-declared Roman Catholics accounted for 95% of the Brazilian population in 1945 and 65% in 2010; in contrast, over the same period, there were growing numbers of Protestants (up from 3% to 22%) and people with no religion people (up from 1% to 8%) and other religions (up from 2% to 5%).^43^ According to the Roman Catholic and Protestant religions, conservative behaviors should be practiced by their followers. Thus, use of illicit drugs is extremely condemned. In this regard, a previous study^44^ showed that religion is a protective factor against drug use. The lower rates of hospitalization due to psychoactive substance use among women than among men may be also associated with the fact that women are more religious than men.

A different profile was seen with regard to mental and behavioral disorders due to psychoactive substances: a slight increase rate among women aged 50 to 59 years was reported in this study, while a steady rate among women aged 60 and older and at all ages among men was observed. The increasing rate among women aged 50 to 59 years may indicate significant distress in this age range. In a previous study,^45^ it was reported that during this period in life, the rates of suicide and suicide attempts among women are higher, and this was correlated with the experience of menopause and some cultural factors that accompany this phase of life among women in Brazil. Hormonal changes can act as facilitators of melancholic processes, and this can be accompanied by a possible feeling of “an end to femininity”, accompanying the loss of reproductive capacity. Menopause, as a symbol of the aging process, can bring sadness, low self-esteem and frustration.

Another external psychological factor that can trigger melancholic feelings at this time in life is the possibility that this may coincide with the phase in which grown-up sons and daughters usually leave the maternal home in Brazil. For women who have taken motherhood as their main objective in life, or who have invested most of their time and energy in this, the departure of their offspring can generate a feeling of intense emptiness.^45,46^

We therefore believe that the sum of these factors can lead to an increase in alcohol consumption and psychoactive substance abuse, which would explain the growth in hospitalization rates for women in this age group.

Some methodological limitations to this study need to be noted: i) our data were acquired from electronic records and, although registration of these records is mandatory, potential for lack of data or incorrect recording may exist; ii) we could not distinguish between the first hospitalization and re-hospitalization episodes; and iii) the recorded data does not include emergency departments, where entries are mostly due to suicide attempts.^47^

## CONCLUSION

Collectively, we present a comprehensive report on hospitalization due to mental and behavioral disorders caused by abuse of alcohol and other psychoactive substances across Brazil, covering the period from 2008 to 2019. This study provides valuable information about hospitalization according to age, sex and year. Decreasing and steady trends of hospitalization due to mental and behavioral disorders caused by use of alcohol among men and women at all ages were demonstrated. Similar trends were reported for all age ranges among men and women aged 60 years and older. In contrast, a slight increase was seen among women aged 50 to 59 years. These data are crucial for planning mental health services targeting older adults and elderly people who are hospitalized due to mental and behavioral disorders caused by use of alcohol and psychoactive substances.
